# Supplementary material for: Active boundary layers in confined active nematics
Source: Nat Commun. 2022 Nov 5;13:6675. doi: 10.1038/s41467-022-34336-z (PMC9637202; doi:10.1038/s41467-022-34336-z)
Supplement: Supplementary file 1 — Supplementary Information [file 41467_2022_34336_MOESM1_ESM.pdf]

SUPPLEMENTARY INFORMATION

Active Boundary Layers in Confined Active Nematics

Jérôme Hardoüin *et al.*

Supplementary Figures

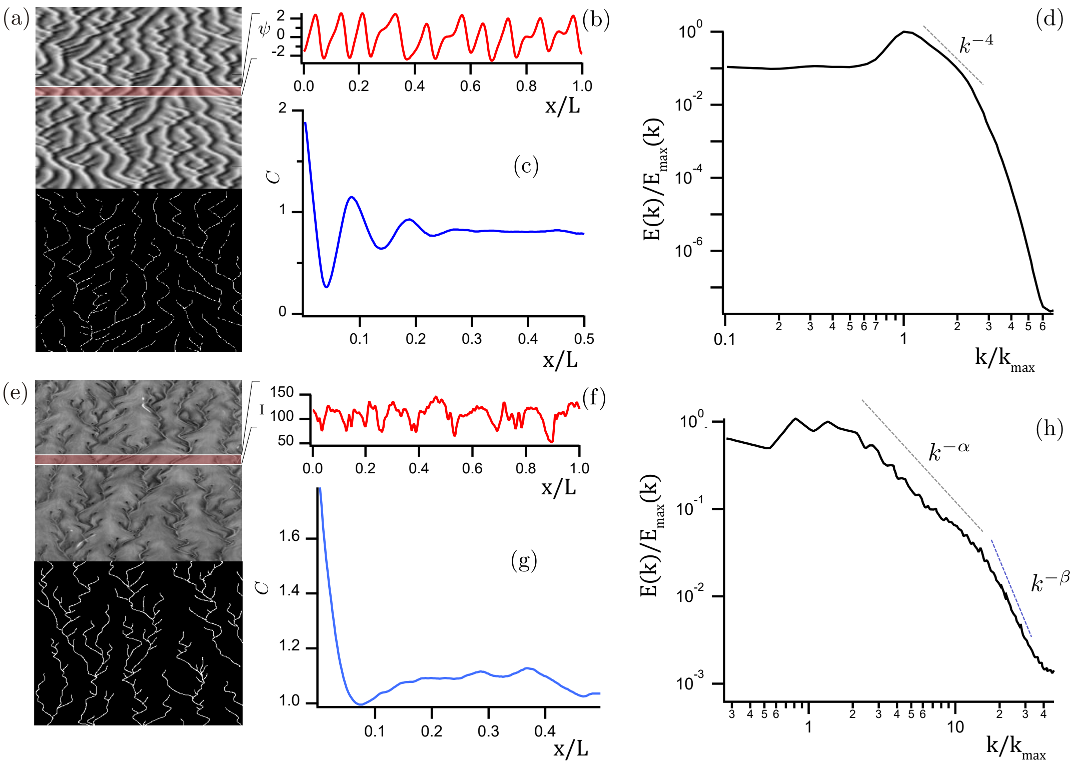

Supplementary Figure 1. Energy spectra from spatiotemporal patterns. (a-d) Panels correspond to the analysis of simulations of the KSE, while (e-h) correspond to the analysis of an ABL. (a,e) Spatio-temporal patterns under study, originated in the phase field ψ of the KSE (a) and in the fluorescence profile of the AN along the inner wall in a 200μm-wide annulus with an inner radius $R_i = 150\mu$m (e). A skeletonized version is overlayed on the bottom half of each kymograph. A sample of the corresponding spatial patterns is shown in (b) and (f). L is the width of the respective kymographs. The time-averaged spatial correlation functions, C, are shown in (c) and (g), and the corresponding Fourier transforms are shown in (d) and (h). The axes are renormalized with the maximum kinetic energy and corresponding wavenumber in the spectra. The expected k^{-4} scaling is shown in panel (d), while two regimes are observed in (h) with $\alpha \sim 1.7$ and $\beta \sim 3.4$.

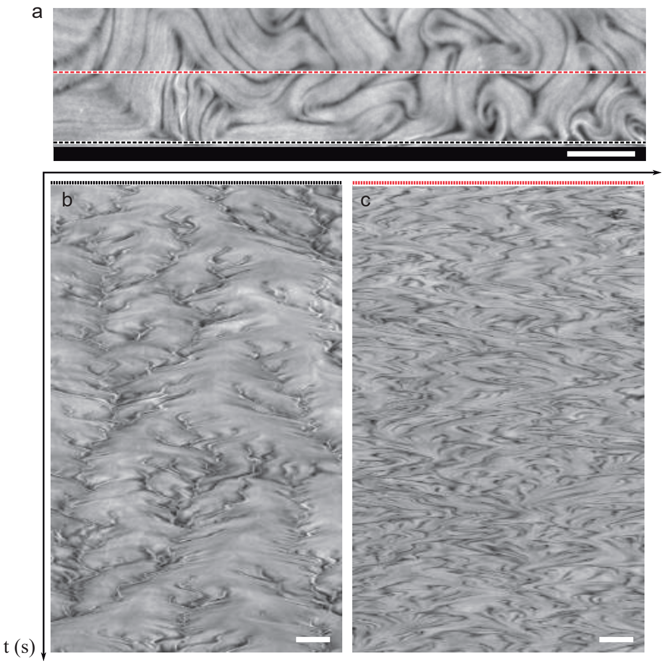

Supplementary Figure 2. Penetration length of wall defects in an active nematic near a flat wall. Scale bars, 100 μm. (a) Fluorescence micrograph. The wall is marked by the black rectangle. The black and red dotted lines correspond to the pixel arrays used to compute the space-time plots of the dynamics close (b) and far (c) from the wall, respectively.

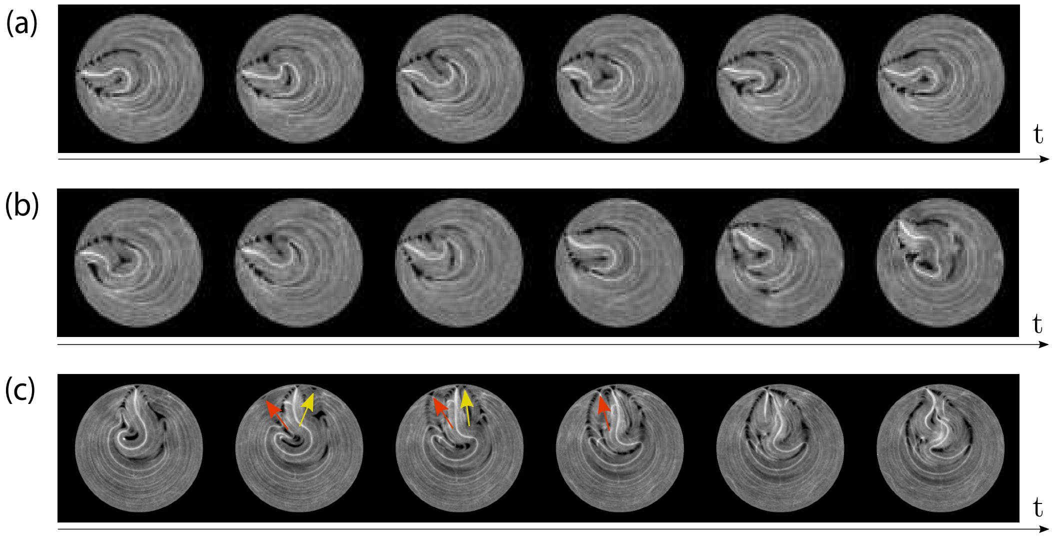

Supplementary Figure 3. Dynamics of isolated wall-defects in a disk. Fluorescence micrograph of active nematics confined inside a disk of 170 μm radius. (a) Time lapse showing transversal fluctuations of a wall-defect with a plume emerging from it. The interval between two frames is 2 s. (b) Time lapse showing the slow drift of the defect along the wall. The interval between two frames is 25 s. (c) Time lapse showing an event with the nucleation of two wall defects followed by their pairwise merging until a single one remains. The newborn defects are pointed to by coloured arrows. The interval between two frames is 2 s.

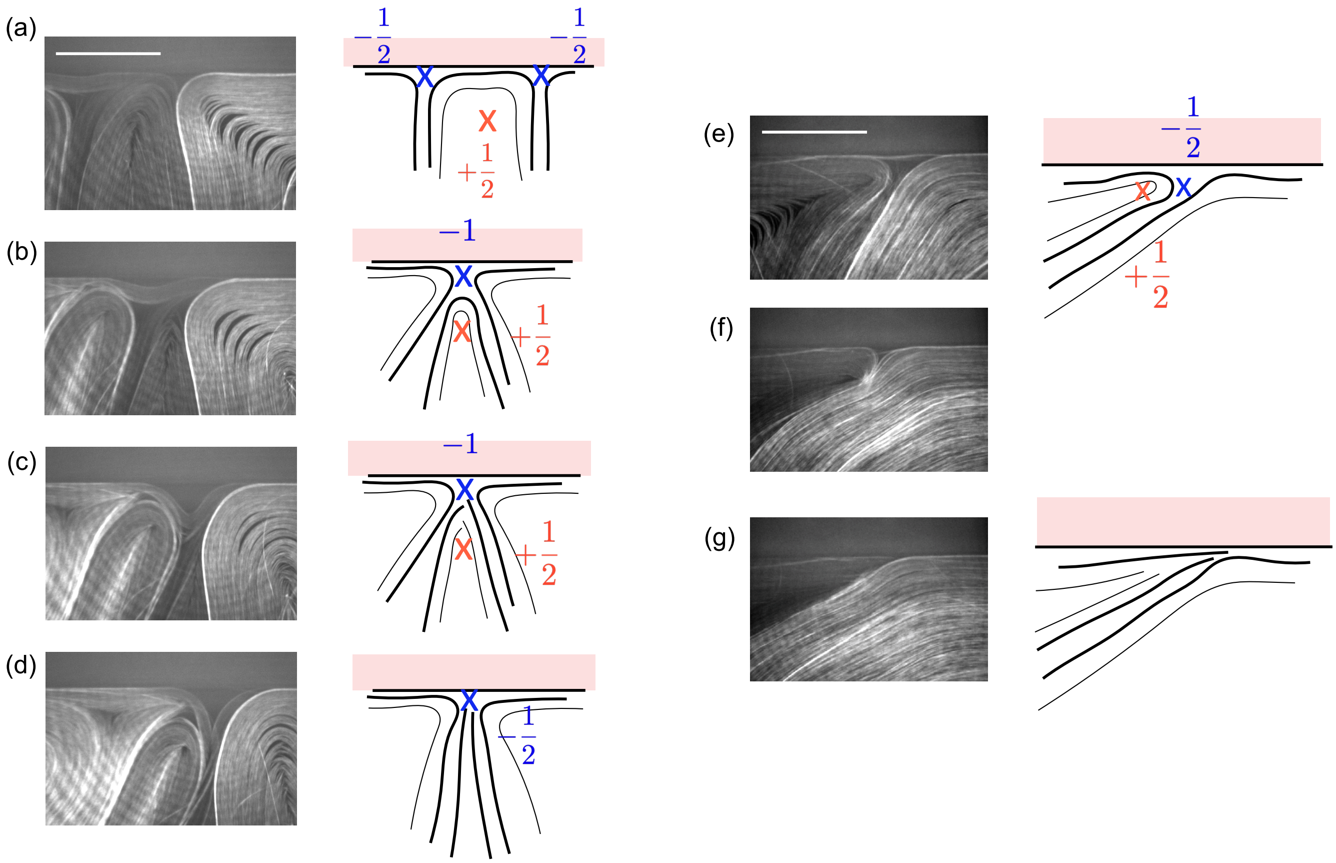

Supplementary Figure 4. Scenarios of wall-defect annihilation near a flat wall. Images are confocal fluorescence micrographs (see also Supplementary Movie 4). A sketch is included to the right of micrographs as a guide for the eye. Each sketch includes the approximate position of defect cores and topological charges. (a)-(d) Recombination of two -1/2 boundary defects with the intervention of a +1/2 defect that forms between them. The recombination of the resulting -1 defect and the +1/2 defect proceeds in a discontinuous manner when the active filaments that form the latter break (b,c) and later reform (d).(e)-(g) Annihilation of a boundary -1/2 defect by merging with a nearby bulk +1/2 defect. These are rare events. Scale bars, 50 μm.

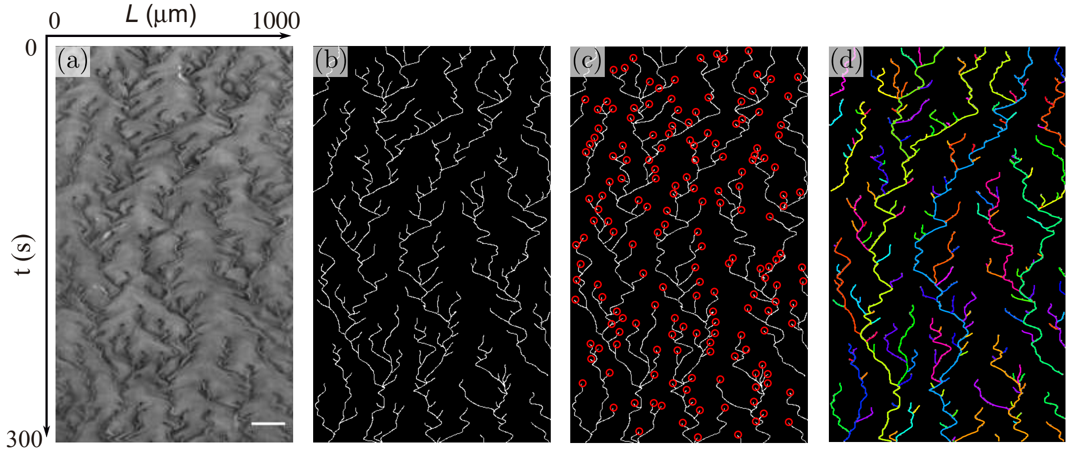

Supplementary Figure 5. Branches and trees in active nematics. (a) Space-time plot of the fluorescence intensity at the inner wall of a 200 μm wide active nematic annulus with inner radius 150 μm. Time goes downwards. Scale bar: 150 μm. (b) Binary skeletonisation of the localized structures in (a). (c) Defect nucleation events, defined as the beginning of a white line in (b). (d) Tracking of defect paths.

Supplementary Notes

Supplementary Note 1: Comparison between the spectral analysis of ABL and KSE simulations

The most distinctive feature of KSE chaos is its spectrum of spatial fluctuations, considered as a proxy of the energy spectrum. It is mainly characterized by a well-defined peak at short wavelengths followed by a short-range scaling at intermediate wave-numbers, k, of the form k^{-4} . Previous publications successfully recovered the shape of KSE spectra from the collective interactions of long-lived fluctuations [1], thereby evidencing the predominant role of these objects in the energy mitigation process [2]. In unconfined ANs, substantial work has recently aimed at elucidating the kinetic energy regulation, so far mostly in simulations [3, 4]. In the vicinity of a boundary, the existence of wall defects and their special collective dynamics will *a priori* result in a different spectrum that should resemble the KSE provided our analogy remains valid for this observable.

As a first approach, we directly compute the spectrum of spatial fluctuations from the fluorescence intensity profile along a wall in an annular channel. We begin by computing the two-point correlation function of the fluorescence intensity at each time, $C(x;t)$, and average it over the duration of the experiment to obtain $C(x)$. Finally, we perform a spectral analysis by computing the Fourier transform of $C(x)$, which is assimilated to the energy spectrum in simulations of the KSE [1]. In Supplementary Figure 1, we compare this analysis performed on simulations of the KSE (see Supplementary Note 2 and Supplementary Figure 1(a-d)) and on experimental fluorescence profiles from the inner wall of an annular channel (Supplementary Figure 1(e-h)).

Qualitatively, KSE and experimental AN spectra do show similarities (Supplementary Figure 1 (d) and (h)), with the existence of a peak followed by a power-law decay. Generic patterns of localized structures, shown in Supplementary Figure 1 (a) and (e), are indeed similar in both cases, albeit detailed textures look somewhat different. Defect-based patterns in the AN seem to contain many more short-lived branches than in the KS-based plots. An excess of such short traces may overshadow the regularity of the tree arrangement, and degrade the spatial correlation, as seen in Supplementary Figure 1 (c and g). Overall, although the detailed collective time-averaged dynamics of wall defects are strikingly similar to that of localized structures in KSE chaos, a more refined experimental study remains to be done to faithfully compare the consequences in terms of energy distribution.

Supplementary Note 2: KSE simulations

The simulation presented in Supplementary Figure 1 (a) was performed using an open source simulation code [5] solving the following equation:

$$\psi_t = -\psi\psi_x - \psi_{xx} - \psi_{xxxx},$$

using periodic boundary conditions on $[0, 32\pi]$ and $\psi(x,0) = \cos(x/16)$ as an initial condition. The computation is based on the Fourier Transform of ψ, $v = \text{fft}(\psi)$. The final plot shown in Supplementary Figure 1 (a) corresponds to $\psi(x,t) = \mathbf{Re}(\text{ifft}(v))$ where ifft is the inverse Fourier Transform operator. It was computed on a discrete domain of $N = 1024$ points and 6000 time steps. For representation in Supplementary Figure 1, only one in six time steps are plotted.

Supplementary References

[1] S. Toh, Statistical model with localized structures describing the spatio-temporal chaos of kuramoto-sivashinsky equation, J. Phys. Soc. Jpn. **56**, 949 (1987).
[2] Y. Pomeau, A. Pumir, and P. Pelce, Intrinsic stochasticity with many degrees of freedom, J. Stat. Phys. **37**, 39 (1984).
[3] L. Giomi, Geometry and topology of turbulence in active nematics, Phys. Rev. X **5**, 031003 (2015).
[4] R. Alert, J.-F. Joanny, and J. Casademunt, Universal scaling of active nematic turbulence, Nat. Phys. **16**, 682 (2020).
[5] E. Renshaw, A solution to the Kuramoto-Sivashinski equation, https://github.com/e-renshaw/kuramoto-sivashinsky.com.
