# Supplementary material for: Active boundary layers in confined active nematics
Source: Nat Commun. 2022 Nov 5;13:6675. doi: 10.1038/s41467-022-34336-z (PMC9637202; doi:10.1038/s41467-022-34336-z)
Supplement: Supplementary file 3 — Description of Additional Supplementary Files [file 41467_2022_34336_MOESM3_ESM.pdf]

Description of Additional Supplementary Files

Supplementary Movie 1. Active nematic confined to a ring-shaped channel. Active boundary layers are observed on the inner and on the outer ring walls.

Supplementary Movie 2. Active nematic confined to a circular pool. A single boundary defect is observed most of the time, determining the flow inside the pool.

Supplementary Movie 3: Active nematic confined to a circular pool. The local charge density is overlaid on the fluorescence images.

Supplementary Movie 4: Confocal fluorescence observation of events in the active boundary layer near a flat wall. (a) Unbinding of a -1/2 (wall) and a +1/2 (bulk defects). (b) Annihilation between a wall (-1/2) and a bulk (+1/2) defect. (c) Recombination of two wall defects (-1/2) mediated by the absorption of a bulk defect (+1/2).

Supplementary Movie 5: Active nematic confined to circular pools of different sizes with an etched indentation (white triangle).
